# Facing the Challenge of Acute Autoimmune Liver Disease: Report of a Case and Review of the Literature

**DOI:** 10.4021/gr2009.05.1295

**Published:** 2009-05-20

**Authors:** Aikaterini Mastoraki, Evaggelia Papantoni, Ioannis S Papanikolaou, Olympia Kotsilianou, Dimitrios Kanakis, George Sakorafas, Michael Safioleas

**Affiliations:** a The Fourth Department of Surgery, Athens University, Medical School, ATTIKON University Hospital, 1 Rimini str., 12462, Chaidari, Athens, Greece; b Department of Gastroenterology and Hepatology, Athens University, Medical School, ATTIKON University Hospital, 1 Rimini str., 12462, Chaidari, Athens, Greece; c University of Athens, Department of Dermatopathology, 75 Mikras Asias str., 11527, Athens, Greece

**Keywords:** Acute autoimmune liver disease, Overlap syndromes, Diagnostic approach, Therapeutic management

## Abstract

Autoimmune liver diseases (ALD) comprise two broad categories: those with a hepatic predominance, autoimmune hepatitis (AIH), and those with a predominance of cholestatic features including primary biliary cirrhosis (PBC) and primary sclerosing cholangitis (PSC). An overlap syndrome (OS) is thought to exist where a patient appears with clinical features of more than one immune-mediated liver disease. Based on one personal observation we report a case of histologically proven OS. The relevant literature is briefly reviewed. A 68-year-old male was admitted in our department with general malaise, anorexia and jaundice. Trans-abdominal ultrasonography as well as computerized tomography showed a slightly enlarged liver with mild fatty deposition and the patient was submitted to transcutaneous liver biopsy. Histological features included inflammatory infiltration of intra-hepatic bile ducts with the presence of endoepithelial lymphocytes and plasma cells, degenerative distortions and even focal damage of relevant epithelial cellular structures, periductal fibrosis, portal inflammation, interface hepatitis, ductular proliferation and canalicular cholestasis. It is apparent that exact definitions pertaining the OS are limited and general consensus on the precise determinations of overlap/variant syndromes is warranted in order to enable more accurate future research and establish common guidelines in the management of the condition.

## Introduction

Autoimmune liver diseases (ALD) include a spectrum of disorders usually defined by a combination of clinical, biochemical, histological and radiological criteria. They comprise two broad categories: those with a hepatic predominance, autoimmune hepatitis (AIH), and those with a predominance of cholestatic features including primary biliary cirrhosis (PBC) and primary sclerosing cholangitis (PSC) [1, 2]. AIH is a progressive inflammatory liver disorder of unknown etiology that is reported to be a consequence of aberrant autoreactivity [3]. Although AIH is generally chronic, acute and fulminate disease presentation has also been described. Immune-mediated hepatitis is characterized by the presence of autoantibodies, abnormally elevated serum transaminase levels and hyperglobulinemia, which is combined with histological characteristics such as multilobular necrosis, interface hepatitis and mononuclear cell infiltration [4]. This disease process has been codified by the International Autoimmune Hepatitis Group (IAIHG), and a scoring system originally intended for research purposes has been widely applied in clinical practice [5]. PBC, in contrast, is characterized by circulating detectable antimitochondrial antibodies (AMA), cholestatic biochemistry and middle-sized bile ductular damage with portal inflammation and granulomas on histological examination. PSC represents a fibro-obliterative cholangiopathy, which may affect bile ducts of any sizes and results in progressive cholestasis and biliary cirrhosis. Unlike other ALDs, PSC mainly affects men and is often associated with ulcerative colitis or other inflammatory bowel diseases.

It is diagnosed in the presence of cholestatic biochemistry, characteristic cholangiographic findings and histopathology, since no specific immunological profile has been confirmed. An OS (OS) is thought to exist where a patient appears with clinical features of more than one immune-mediated liver disease. Although they may fulfill diagnostic criteria for both entities, this, however, is not essential, the attributes of two diseases may occur simultaneously, or indeed the patient may have features of one disease at one point of time and develop characteristics of another disorder at a later date. Reports to date have identified a range of definitions with corresponding incidences. Nevertheless, no established clinical diagnostic criteria have been proposed for OS, its pathogenesis is not elucidated and relevant surveillance remains unclear.

Based on one personal observation, we report a case of histologically proven OS. The relevant literature is briefly reviewed. Clinical course, long-term outcome and the histologic pattern of the above mentioned disorders are discussed, while the role of current specific radiographic findings in the early diagnosis and therapeutic management of this serious and potentially lethal disease category are emphasized.

## Case report

A 68-year-old male was admitted in our department with general malaise, anorexia and jaundice over the preceding 6 weeks. He did not describe similar episodes of gastrointestinal abnormalities or abdominal discomfort such as epigastralgia or pain in the right upper quadrant. He presented with a past medical history of partial gastrectomy (Billroth Type I) for gastric cancer 6 years ago, haemorroidectomy 12 years ago, and appendectomy in childhood. He also referred intermittent episodes of hypertension. Meticulous clinical examination revealed a palpable liver edge and subicterus of the sclera combined with a recrudescence of symptoms of anemia. Hematological analyses demonstrated a hemoglobin level of 11.2 g/dl, total bilirubin 22.27 mg/dl, conjugated bilirubin 12.98 mg/dl, ALT 2681 U/L, AST 1615 U/L, GGT 172 U/L, ALP 185 U/L, LDH 980 U/L and INR 2.22. Markers for hepatototropic viruses showed positive IgG and negative IgM levels for CMV and positive anti-HAV antibodies. Further laboratory examination revealed antinuclear antibodies (ANA 1: 80) and smooth muscle antibodies (SMA 1: 60) positivity. AMA, p-ANCA, c-ANCA and liver kidney microsome (LKM) antibodies detection proved negative. Markers for infectious diseases such as ESR and CRP were elevated and tumor factors elucidated increased carboanchidratic CA 19-9 antigen (141.9 U/mL) and AFP (7.3 ng/mL) titers. All other indices as well as plain chest and abdominal X-rays were within normal limits. Potential etiologies for other chronic liver diseases were excluded by medical history, serum seruloplasmin, transferring saturation and alpha-1 antitrypsin levels. Signs of overt ascites, oesophageal varices and concomitant autoimmune liver disorder including diabetes mellitus, thyroid disease, rheumatoid arthritis and ulcerative colitis were absent.

Trans-abdominal ultrasonography (U/S) as well as abdominal Computerized Tomography (CT) showed a slightly enlarged liver with mild fatty deposition and normal gallbladder appearance without multiple hyperechoic lesions suggestive of the presence of calculi. Additional parenchymal hepatic abnormality, which was combined with focal defect or dilation of either extra or intra hepatic biliary tract, was not confirmed. Disease recurrence was excluded by subsequent endoscopic evaluation, which was restricted to the estimation of the upper gastrointestinal tract without the contribution of Endoscopic Retrograde Cholangiography (ERCP) due to the patient’s previous surgical intervention. As the endoscopic bioptical sampling proved insufficient to establish the diagnosis, the patient was transferred to the interventional radiologic department and submitted to transcutaneous liver biopsy. During the procedure, two adequate specimens were obtained and sent for histological examination.

**Figure 1:**
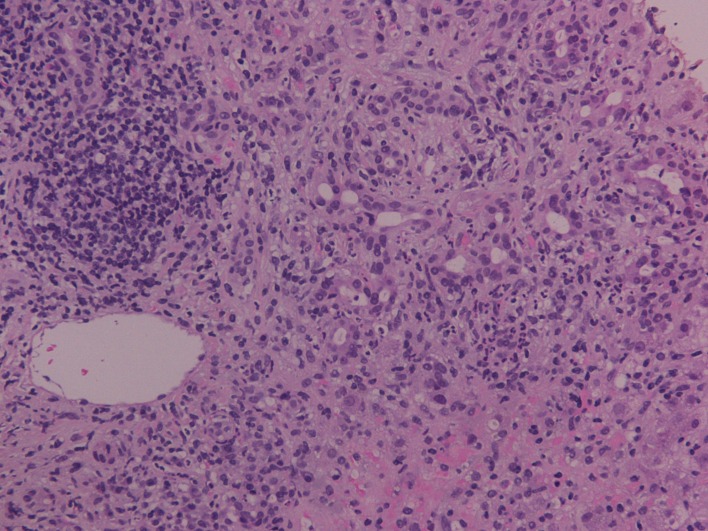
Inflammatory infiltration of intra-hepatic bile ducts with the presence of endoepithelial lymphocytes and plasma cells, periductal fibrosis, portal inflammation and canalicular cholestasis (H-E stain, × 200).

Samples were estimated by a dedicated pathologist and stains for hematoxyline-eosin (H-E), connective tissue (Masson trichrome), iron and copper-associated protein were available. Recorded histological features included inflammatory infiltration of intra-hepatic bile ducts with the presence of endoepithelial lymphocytes and numerous plasma cells, eosinophils and neutrophils, degenerative distortions and even focal damage of relevant epithelial cellular structures, periductal fibrosis, portal inflammation, interface hepatitis, ductular proliferation and canalicular cholestasis (Fig. 1). Additional analysis also demonstrated signs of piecemeal necrosis in liver parenchyma, whereas, many hepatocytes appeared with a hydropic degeneration of their cytoplasm (ballooning), thickening of their cytoskeleton, formation of Mallory particles, lymphocyte resetting and modular or severe periseptal lobular inflammation. Finally hyperplastic biliary vessels with indications of pericholangitis were apparent and compatible with an ALD of the type of OS. The patient was transferred to the department of hepatology where the administration of prednisolone at 0.5 mg/kg/day was implemented. The steroid dose was subsequently individually decreased to the lowest required to reassure biochemical remission, and the patient was sustained partial clinical recovery for at least 3 months on maintenance therapy.

## Discussion

AIH was first described by Waldenstrom in 1950 as a liver disease affecting young women and its diagnosis continues to be a challenge for clinical hepatologists. The IAIHG proposed several simplified diagnostic criteria, which were revised in 1999 [6] and objectively identified patients with either ‘definite’ or ‘probable’ AIH (Table 1) [7]. The majority of patients exhibited clinical and histological features of chronic hepatitis or liver cirrhosis. In our case, an increased serum value of transaminase combined with hyperbilirubinemia and concomitant evidence of interface or lobular hepatitis, plasma cells infiltration and rosette formation were observed. Multinucleated giant cells are usually obvious, and hepatocytes in the periportal area are prone to cell division and repair during degenerating process. Therefore, similar cellular structures were recognized in our case identifying potential active inflammation around the central vein. The mechanism of cell fusion is also proposed to result from membrane instability due to microtubular defects. In accordance with recent studies, CK-19 positive cholangiolar cells are subjected to proliferation process while immature cellular elements compensate for parenchymal damage and further destruction in the portal tract. Differentiation into hepatocytes or bile ducts could not be excluded. Nevertheless, the confirmation of peripheral biliary injury in association with the presence of autoantibodies indicating destructive cholangitis contributed to the rejection of the diagnosis of AIH, even though liver functional disturbance was improved by the administration of prednisolone. Thus, in each case of AIH, severe inflammation or massive necrosis in the periportal level could influence the bile duct structures, but whether associated injury is related to the variant type should be investigated by serial liver biopsies before and after glucocorticoid administration. In addition, the diagnosis of AIH is often complicated by the presence of confounders, such as alcohol abuse, viruses or drugs, and there is insufficient evidence that any of the above mentioned autoantibody categories are directly involved in the pathogenesis of the disease. Therefore, well established surveys asserted that the diagnostic definition of AIH requires the examination of several bioptical samples performed in our patient.

**Table 1:** Simplified diagnostic criteria for autoimmune hepatitis

Variable	Cutoff	Points
ANA or SMA	≥ 1:40	1
ANA or SMA	≥ 1:80	
or LKM	≥ 1:40	2*
or SLA	Positive	
IgG	> Upper normal limit	1
	> 1.10 times upper normal limit	2
Liver histology (evidence of hepatitis is a common condition)	Compatible with AIH	1
	Typical AIH	2
Absence of viral hepatitis	Yes	2

ANA: Antinuclear antibody; SMA: Smooth muscle antibody; LKM: Liver/Kidney microsome type; SLA: Soluble liver antigen. Resulting in: ≥ 6: Probable AIH and ≥ 7: Definite AIH. *Addition of points achieved for all autoantibodies (maximum, 2 points).

**Figure 2:**
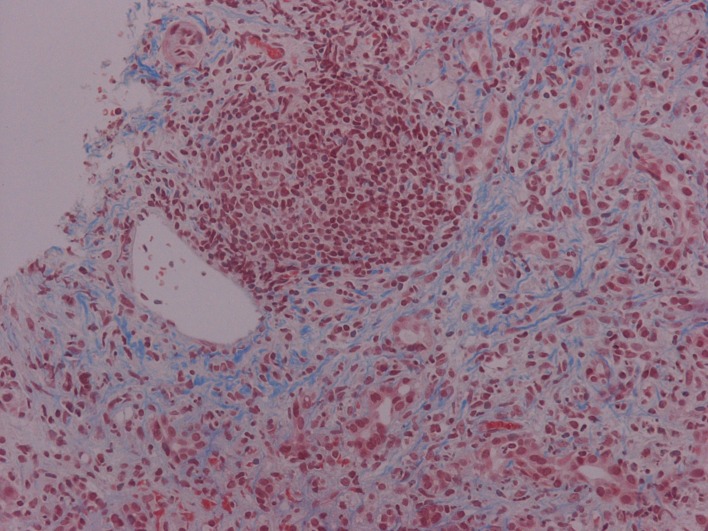
Chronic cholestatic disease characterized by progressive inflammation and fibrosis of intra- and extra-hepatic bile ducts (Masson trichrome stain × 200)

PSC is a chronic cholestatic disease, of unknown etiology, characterized by progressive inflammation and fibrosis of intra- and extra-hepatic bile ducts (Fig. 2) [8]. The diagnosis is based on the cholangiographic parameters obtained by ERCP. Depiction of irregularly distributed multifocal stenoses and dilations involving the intra- and extra- hepatic biliary tract has been recorded [9]. Simultaneous co-existence of inflammatory bowel disease has been well documented, while ulcerative colitis remains the most common ultimate manifestation of PSC. In our case, potential association of gastrointestinal inflammation was assayed by conventional endoscopic criteria. Patients presented with parallel co-morbidity of PSC and bowel inflammation are prone to develop colorectal carcinoma as well as cholangiocarcinoma. Neoplastic complications affecting the liver and digestive tract constituted essential risk factors to reduce survival in patients with PSC and were absent in our case. In spite of several recently published time-fixed Cox regression prognostic models, estimation of outcome for the individual PSC patient still remains a challenge. Moreover, at present, it is difficult to recommend a useful strategy for preventing potential neoplastic development despite routinely monitoring of serum tumor markers. Finally, in our patient, increased carboanchidratic CA 19-9 antigen and AFP titers were not associated with malignant potential of hepatic lesions.

The term “overlap” syndrome has been implemented since two decades to describe the concurrence of clinical, biochemical, serological and histological features reminiscent of AIH, PBC, and/or PSC [10]. Similar disorders are determined as conditions with both hepatic and cholestatic elements and usually present a progressive course toward liver cirrhosis and hepatic failure without prompt adequate treatment [11]. The AIH/PBS OS remains the most common form, affecting almost 10% of adults with AIH or PBC. The AIH/PSC OS is predominantly encountered in children, adolescents and young adults with AIH or PSC [12]. There is no significant difference in the acuity of symptom onset or time to diagnosis among the patient groups. The most common presenting sign is jaundice in both AIH and PSC/AIH overlap patient categories; whereas patients with PBC/AIH overlap most frequently appear with general malaise and lethargy. Additional combined features of PSC and AIH include pruritus, stigmata of chronic liver disease, abdominal distension, upper gastrointestinal bleeding, ascites and encephalopathy [13]. The mode of onset of AIH-PSC OS may be acute, insidious or asymptomatic. In our case, laboratory findings consisted mainly in hyperbilirubinemia and elevated serum transaminase levels.

OSs are detected most reliably on grounds of serologic markers and histology. Liver autoantibodies are crucial for the correct diagnosis and classification [14]. The revised scoring system for the diagnostic approach of AIH is calculated according to the latest report of IAIHG. The diagnosis of AIH-PSC OS is established when the following criteria are met: (i) a total aggregate score for AIH > 15 (defining the AIH as “definite”); (ii) ANA or SMA present in a titer of at least 1: 40; (iii) liver histology with piecemeal necrosis, lymphocyte resetting, moderate or severe periportal, or periseptal lobular inflammation [15]. LKM and AMA titers are commonly within normal limits. In addition, cholangiography as well as ERCP are often warranted to distinguish between PSC with an autoimmune serology profile of AIH and AIH as a distinct clinical entity [16]. Semi-quantitative histological assessment of the severity of chronic hepatitis should always be performed in accordance with standard methodology evaluating necroinflammatory activity and the extent of fibrosis. Biopsy is typically repeated in cases of occurrence of relapse or lack of response to conventional therapy. A concomitant rheumatologic autoimmune condition including juvenile rheumatoid arthritis, seronegative spondilitis and peripheral vasculitis is often described.

Although it has been suggested that the two disorders may be sequential in their appearances, clear clarification of evolution has not been adequately documented in adults. Therefore, patients with well-established AIH can, after variable illness duration, develop PSC [17]. The majority of published studies refer to AIH/PSC OS patients treated with corticosteroids or combination with azathioprine. Possible efficacy of cyclosporine has also been tested. Immunosuppression combined with ursodeoxycholic acid (UDCA) seems to be beneficial, but cannot prevent long-term progression toward cirrhosis. Therapy with UDCA has been proposed to be associated with improvement in abnormal serum biochemical liver parameters in patients with PSC without consistent amelioration in hepatic inflammation, fibrosis or histological stage of the disease [18]. During treatment, obstruction of major ducts may lead to deterioration of liver function, which could be prevented by endoscopic dilation of the stenotic lesions [19]. Further improved response to therapy with combined drug administration versus either UDCA or steroid monotherapy in patients with AIH/PSC has been recently supported by large tertiary referral centers. As far as liver transplantation (LT) is concerned, the course and outcome of patients after the surgical intervention for PSC are still debated. Allograft autoimmune liver disease was observed in 43% of long-term post-LT PSC patients and native liver hilar xanthogranulomatous cholangiopathy negatively impacted post-LT graft and patient survival [20]. Furthermore, increased incidence of malignancies in the non-recurrent group may reflect potential over-immunosuppression.

In conclusion, AIH/PSC OS is a variant of PSC that mainly affects young people and should be suspected in the case of little response to immunosuppression in a patient suffering from AIH. However, since late sequelae have been described, a careful monitoring of the biochemical markers of cholestasis is highly recommended. A cholangiographic study is also implemented in patients presenting histological characteristics of AIH and associated ductular damage. Furthermore, the survival of patients with PSC-AIH is significantly lower compared to those presenting either AIH or PBC-AIH features. It is apparent from the literature that exact definitions pertaining the OS are limited and general consensus on the precise determinations of overlap/variant syndromes is warranted in order to enable more accurate future research and establish common guidelines in the management of the condition.
